# Experiences of nursing students regarding clinical placement and support in primary healthcare clinics: Strengthening resilience

**DOI:** 10.4102/hsag.v26i0.1615

**Published:** 2021-10-29

**Authors:** Beauty M. Zulu, Emmerentia du Plessis, Magdalena P. Koen

**Affiliations:** 1 Department of Nursing, Faculty of Health Sciences, Ann Latsky Nursing College, Johannesburg, South Africa; 2 NuMIQ Research Focus Area, Faculty of Health Sciences, North-West University, Potchefstroom, South Africa

**Keywords:** clinical placement, experience, nursing students, primary healthcare, resilience

## Abstract

**Background:**

This research addressed the need to have a deeper understanding of nursing students’ experiences of clinical placement and support in primary healthcare (PHC) settings.

**Aim:**

To explore and describe the experiences of nursing students’ clinical placement in PHC settings.

**Setting:**

The study was conducted at PHC clinics in a specific province in South Africa.

**Method:**

A qualitative, exploratory, descriptive and contextual research design was used. The population comprised fourth-year nursing students selected using purposive sampling. Five semi-structured focus group interviews were conducted. Data saturation determined the sample size which comprised 25 nursing students. Thematic data analysis produced four main themes.

**Results:**

The main themes were (1) the meaning of placement, (2) experiences of clinical placement, (3) needs for support, and (4) resilience of nursing students.

**Conclusions:**

Nursing students encountered negative and positive experiences. Both experiences confirmed that PHC settings can be valuable learning opportunities for nursing students. Nursing students appreciated the supervision of professional nurses who created an atmosphere conducive for teaching and learning by welcoming and encouraging nursing students’ independence. Recommendations include: appointing a professional nurse specifically for supervision of nursing students, tutor support before summative examinations, improvement of services, including the availability of resources.

**Contribution:**

This article contributes to awareness of how nursing students manage to stay resilient irrespective of negative experiences during clinical placement and how tutors and professional nurses can support them. The study’s recommendations can guide professional nurses, nurse educators, nurse managers and other healthcare PHC workers to support nursing students.

## Introduction

The focus of primary healthcare (PHC) is on the health needs of clients at all levels, including preventive, promotive, curative, and rehabilitative levels of care (Robertson et al. 2016). All PHC clinics should offer comprehensive services, which are available, accessible, affordable, effective and efficient thereby bringing health closer to communities. Primary health care forms an integral part of a country’s health system, and it is the main focus that constitutes the first element of a continuing healthcare process (Druetz 2018).

Nursing Education Institutions (NEI) in South Africa (SA) place nursing students at PHC clinics to obtain clinical learning experience. These clinical environments should have a positive learning atmosphere so that nursing students are able to transfer classroom knowledge to clinical practice (Motsaanaka, Makhene & Ally 2020). A positive clinical learning environment includes cooperation between staff members, a positive atmosphere and an environment where nursing students are regarded as colleagues, and they must be able to acquire significant information and respond appropriately to patient care situations (Chiona 2020).

Clinical learning for nursing students at PHC clinics requires adequate support from the NEI and PHC nurses (Mekgoe et al. 2019). The role of PHC nurses becomes imperative when looking at support for nursing students placed at PHC clinics for clinical practice (Barbiani, Dalla Nora & Schaefer 2016). The roles of PHC nurses include organising staff and equipment, patient care, teaching and learning of nursing students. Primary health care nurses should guide and support nursing students during clinical practice. All qualified professional nurses require training especially on tuberculosis (TB), human immunodeficiency virus and acquired immunodeficiency syndrome (HIV and AIDS) (Mekgoe et al. 2019), as well as maternal and child health (Rispel & Bruce 2015) to be able to transfer the newly acquired knowledge and support to nursing students.

Placement at PHC settings provides training opportunities for nursing students, as well as opportunities to develop responsibility, self-confidence and resilience – namely courage to endure difficult situations (Fröberg et al. 2018). Positive clinical learning experiences that build resilience include orientation to the service, a sense of belonging to the PHC team, independent working opportunities and growth towards professionalism (Robertson et al. 2016). Nursing students also experienced challenges during clinical practice placements such as shortages of human and material resources, staff burnout, ineffective quality control, inadequate continuing education and a lack of support and mentoring from professional nurses who might regard nursing students as part of the PHC ‘workforce’ (Robertson et al. 2016:423). These challenges require nursing students to be resilient, ready and prepared to cope with such situations. (Robertson et al. 2016:423). Therefore, this article aimed to explore and describe the experiences of nursing students’ clinical placement in PHC settings and to formulate recommendations to support and strengthen students’ resilience during challenging situations.

### Research problem

Clinical practice placement at PHC clinics requires nursing students to be resilient to be able to grow and cope (Lopez et al. 2018). On the one hand, PHC clinics as clinical learning environments offer valuable opportunities for nursing students to apply theoretical knowledge and to develop into competent, independent, professional nurses (Mackey et al. 2018; Phafoli et al. 2018). On the other hand, nursing students may experience challenges that require them to be resilient, such as lack of resources and lack of support (Thomas & Asselin 2018). Professional nurses who work at PHC clinics can play a valuable role in guiding nursing students (Barbiani et al. 2016).

There was a limited research on nursing students’ experiences in PHC clinics in a province in SA, and how professional nurses working at PHC clinics can support nursing students. This research was important in the context of a province with the highest numbers of NEI, with equally high numbers of students (SANC 2018), and consequently high numbers of students who need to be placed at PHC clinics for clinical practice. For example, at the time of this study, there were 197 fourth-year nursing students registered at a specific public nursing college who needed clinical placement at PHC clinics. Literature confirms that the need for research on the resilience of professionals involved in PHC remains evident (Robertson et al. 2016). At the time of the research, the first author’s own experience as a professional nurse in a PHC clinic also confirmed the need for this research. Whilst more recent research has also been reported at the international level and in the African context on the learning experiences of nursing students during placement at PHC clinics, support for nursing students during clinical placement in PHC remains lacking (Fröberg et al. 2018; Mackey et al. 2018; Mbakaya et al. 2020). There is thus an ongoing need for research to explore and describe nursing students’ experience of clinical placement in PHC clinics, in this case in a province in SA, and to apply the findings to recommend strategies for support to strengthen students’ resilience during challenging clinical situations.

The following research questions were based on the problem statement:

- What are the experiences of nursing students’ clinical placement in PHC settings for clinical nursing practice?
- How can nursing students be supported to strengthen their resilience during challenging situations at clinical placements in PHC settings?

### Purpose

The purpose of the study was to explore and describe the experiences of nursing students’ clinical placement in PHC settings and to formulate recommendations to support and strengthen students’ resilience during challenging clinical situations.

### Definitions of keywords

**Clinical practice –** involves the development of knowledge, skills and attitudes by correlating theory and practice, enabling an individual to perform fully in the process of becoming a nurse and a professional, without putting patients at risk. It includes not only the performance of nursing procedures but the relationships with patients and colleagues (Chiona 2020). In this study, clinical practice referred to nursing students’ learning experiences encountered at PHC clinics.

**Experience –** a hands-on job that prepares nursing students for a diverse patient care environment in a variety of settings, and it provides a vast wealth of knowledge. It includes planned experiences for a specific nursing education course and experiences gained by nursing students in a hospital, clinic, healthcare centre, and the community (Rajeswaran 2016). This study focused on the experiences of nursing students of clinical practice in PHC clinics.

**Nursing students** – student nurses registered under Regulation 425 of the *Nursing Act* (Act 33 of 2005) as amended to qualify as a nurse (General, Psychiatric, Community) and Midwife (South African Government 2005). In this study, nursing students referred to the fourth-year nursing students enrolled at a public nursing college for a nursing diploma who had been allocated to specific PHC clinics for acquiring clinical practice learning opportunities.

**Primary healthcare** – is essential healthcare based on practical, scientifically sound and socially acceptable methods and technology. This healthcare service is made accessible and affordable to the community to maintain their health and development (Druetz 2018). In this research, the focus was on municipal PHC clinics in a specific region of a South African province.

**Resilience** – the ability to recover one’s strength, health, energy, spirit and motivation after experiencing a setback or adversity (Robertson et al. 2016). In this research study, the focus was on the resilience of nursing students to cope with challenges encountered during their clinical practice assignments at PHC clinics.

## Research design and methods

A qualitative, exploratory, descriptive and contextual research design was used to explore the experiences of nursing students’ placement in PHC settings for clinical practice (Botma et al. 2015). This design was chosen as participants were studied in a natural context. Also, this design was in line with the purpose of the study, namely to explore and describe nursing students’ experience (Botma et al. 2015). The research method is presented next by discussing the study setting, study population and sampling strategy, data collection, and data analysis.

### Research setting

The study was conducted at PHC clinics in a specific province in SA, administered by the local government, also called municipal clinics. These clinics comprise: 14 fixed clinics and 1 fixed porta cabin. A porta cabin is a temporary, flexible building designed to be movable where accommodation is required for an uncertain period. The PHC clinics provide comprehensive services, such as ante-natal services, child health and immunisation, reproductive health and family planning, adolescent and youth-friendly services, acute and chronic services. The clinic with the highest workload of patients reports statistics of 15 000–17 000 patients per month and the clinic with the lowest reports 800–1000 patients per month. The average staff starting with the busiest clinic consists of eight professional nurses, two enrolled nurses and two auxiliary nurses and other support staff members. The least busy clinic has four professional nurses and one auxiliary nurse. Four to six fourth-year nursing students from a specific public nursing college had been placed on several occasions at these PHC clinics from their second year. The placement usually takes 4–6 weeks. Each professional nurse would supervise a maximum of two nursing students per consultation room.

### Study population and sampling strategy

The population comprising 197 fourth-year nursing students was identified from a public nursing college’s records. Purposive sampling (Brink, Van der Walt & Van Rensburg 2018) was used to select participants whose age ranged between 22 and 30 years. The inclusion criteria required that participants be fourth-year nursing students regardless of age or gender, familiar with PHC clinical settings, and able to communicate in English. Students were invited and requested to consent voluntarily to participate in the study. Sampling resulted in five focus groups (Polit & Beck 2021) comprising five to eight participants each. The total number of participants was 25. Data saturation determined the sample size.

### Data collection

Five focus group interviews including the pilot study were conducted between June and September 2014 at five different PHC clinics where the participants were placed. Looking at more recent, similar research findings (Motsaanaka et al. 2020), the findings of this research are still relevant. More recent studies still report that students need emotional, cognitive and systems support such as personal attention, care, guidance to gain confidence and socialisation in nursing (Bazrafkan & Kalyani 2018; Hosseinabadi et al. 2020; Hugo, Botma & Raubenheimer 2018; Kgafele, Coetzee & Heyns 2015; Mbakaya et al. 2020; Mekgoe et al. 2019), whilst the current study offers valuable findings and recommendations that can add to the body of knowledge on this important topic.

Semi-structured focus group interviews were used to explore nursing students’ experiences regarding their placement at PHC for clinical practice. Interview questions were developed in line with the research problem and purpose: ‘What does it mean to you to be placed in PHC clinics for clinical practice?’;, ‘Describe your positive, supportive and helpful experiences during clinical practice at PHC clinics?’; ‘Please elaborate further on how you can be supported to cope with challenges?’ and ‘What enables you to cope irrespective of challenges you encountered?’

The first author did a role-play exercise to practise interviewing and recording skills before the actual data collection was done. A trial run focus group interview was also done to test the appropriateness and applicability of questions, which were assessed by the study supervisors. The trial run focus group interview entailed that the first author conduct and audio-record the first focus group interview with the first group of participants, and held a discussion with the supervisors to reflect on the appropriateness and clarity of the interview questions. No changes were needed from the interview questions and further focus group interviews were continued. Each focus group interview lasted for approximately 60 min. The challenges experienced were that the venue for the interviews had to be changed from the nursing college to PHC clinics because of the tight lecture time table and block tests that were scheduled for nursing students.

Immediately after each focus group interview, field notes were taken, and recordings were checked for audibility and completeness, and transcriptions were compiled by the first author (Botma et al. 2015). Data saturation was reached after five focus group interviews, including the pilot focus group interview, each attended by five to eight participants.

### Data analysis

Thematic data analysis (Gray, Grove & Sutherland 2017) was used. Manual analysis was undertaken. Transcriptions of interviews were read carefully, supported by detailed notes, to get a sense of the whole data set. One transcript was then selected and read to extract important ideas through underlining central phrases. This process was repeated with the remaining transcripts. The underlined words and phrases were grouped into main categories, sub-categories and leftover categories, which were then grouped into themes and sub-themes.

### Trustworthiness

Five epistemological standards of trustworthiness: truth value, applicability, consistency, neutrality and authenticity were used in this research (Guba and Lincoln, cited by Botma et al. 2015). This included strategies to ensure truth value, namely credibility strategies, including prolonged interaction with the participants and peer examination of the research proposal and dissertation. Prolonged interaction was ensured by building rapport with the interviewees, spending 2 h at the nursing college during an information sharing session and another 2 h at PHC clinics to familiarise oneself with the cultural values and eliminate misconceptions. Furthermore, time was given to participants before the commencement of interviews to ask questions and allay anxiety. Strategies to ensure applicability included a thick description of the research design and methods to ensure transferability. To ensure consistency through the strategy of dependability, the same strategy of a thick description of the research was followed, for example, comprehensive documentation such as transcribed audio-recorded interviews, results, conclusions, limitations and recommendations were explained and thorough transcription of interviews of each participant, to ensure that an audit trail is possible. Neutrality was ensured through confirmability, namely by involving a co-coder during data analysis and through research supervision. Authenticity further contributed to trustworthiness, namely that the exact words of the participants as recorded and transcribed formed the data set for the research.

### Ethical considerations

Ethical clearance to conduct the study was granted by the Human Research Ethics Committee of the Faculty of Health Sciences, North-West University (reference number: NWU-00036-11-A1). Permission to conduct the study was also granted by the campus head of the public nursing college and by the city manager and the research committee at the district office of PHC clinics. The first author worked as an operational manager of a PHC clinic, which serves as a community nursing science placement for nursing students involved the mediator to manage power relations. The mediator was present during information sharing sessions and she co-ordinated the signing of consent forms in the absence of the first author. The mediator later provided the first author with a list of potential participants.

The principle of beneficence was upheld by recognising that the participants could potentially experience psychological distress when describing their personal views about their experiences in clinical practice at PHC clinics. The first author thus made arrangements with a social worker to counsel nursing students should a need arise. One participant cried during interviews and was offered to be referred to the social worker, but she chose not to make use of the offer. The principle of respect was upheld through providing freedom of choice to participate as indicated in information leaflets with objectives, purposes and benefits of the study and obtaining informed consent for participation and audio-recording. No participant was manipulated or forced to participate in the study. The principle of justice was upheld by selecting participants according to the selection criteria. The participants were made aware that only partial confidentiality could be ensured, and they were requested to set their own ground rules regarding upholding confidentiality. Privacy was ensured as interviews were conducted in a private room. Furthermore, transcripts of the interviews are secured in a safe place.

## Results

Four main themes emerged from the semi-structured focus group interviews, with related sub-themes (see Table 1). In the following section, quotes from the focus group interviews are identified by focus group interview (F1, F2, F3, F4, trial run), gender (Female [F], Male [M]), participants (P1, …) and years of age (e.g. 26 yrs). This presentation of the results is followed by a discussion of the results in light of considering existing literature.

**TABLE 1:** Themes and sub-themes.

Theme 1: The meaning of placement at PHC clinics for clinical practice	Theme 2: Positive, supportive and helpful experiences at PHC clinics	Theme 3: Support to cope with challenges	Theme 4: Resilience of nursing students
Gaining experience, knowledge and exposure	Staff-related experiences	Educational support	Passion for the nursing profession
Community-related interaction	Staff-related challenges	Support from professional nurses at PHC settings	Personal strengths
Reach educational objectives	Environment and service-related experiences and challenges	Service and environmental aspects	Support
Clinical guidance	Patient-related challenges		
	Lack of tutor support/supervision		

*Source*: Zulu, B.M., 2015, *Nursing students’ experience of clinical practice in primary health care clinics*, Masters dissertation, North-West University, Potchefstroom, viewed from https://repository.nwu.ac.za/bitstream/handle/10394/15830/Zulu_BM_2015.pdf?sequence=1&isAllowed=y.

PHC, primary health care.

### Theme 1: The meaning of placement at primary healthcare clinics for clinical practice

The participants mentioned that being placed at PHC clinics for clinical practice is to gain practical experience, knowledge and exposure, community-related interaction, to reach educational objectives and to receive clinical guidance. These sub-themes are discussed in detail in the following section.

#### Gaining experience, knowledge and exposure

Being placed at PHC clinics for clinical practice is to gain practical experience, knowledge and exposure to the clinical environment:

‘Gaining the skill to see the patient.’ (F3, F, P2, 22yrs)

Participants stated that the meaning of being placed for clinical practice is to correlate theory to practice. They viewed the clinical practice as putting what they have learned in class into practice:

‘We take what we have learned at school and we incorporate it, and learn how to do it practically, that is what we do.’ (F3, F, P2, 22 yrs)

Participants experienced that the knowledge and skills they gain enrich them with the ability to assess, diagnose and be able to treat minor ailments that the community might have. Furthermore, gaining experience, knowledge and exposure helps them to grow in their profession.

The participants also emphasised that clinical practice in PHC clinics exposed them to comprehensive services:

‘[… *A*]s also the PHC it is not focusing only on one thing it’s a comprehensive care so meaning that whatever the patient is having we nurse the patient holistically before the patients can be referred.’ (F4, M, P2, 23 yrs)

They could implement these services starting with preventative care before patients reach the second level of care, that is the hospital.

Clinical practice exposes nursing students to interact with patients from diverse backgrounds and enable them to learn more by interacting with people from different age groups, genders and cultures:

‘We also learn how to deal with the different people, different cultures … different cultures in regards to our age, us being females, however that regards age, gender, culture, different backgrounds and learning how to deal with such.’ (F3, F, P2, 22 yrs)

#### Community-related interaction

According to the participants, clinical practice helps one to understand the community, that is, the catchment area, their needs, community dynamics and the common diseases that they face. They further stated that clinical practice is about understanding or learning about the community, its health needs and profile:

‘I think they have said it right but in addition I think it also helps me personally mam, maybe to understand the community and their needs and maybe to come with ideas on how to improve, to help the community.’ (F1, F, P3, 24 yrs)

They mentioned that clinical practice in PHC provides opportunities to work with the community and encourages community participation and involvement by means of community-based outreach activities to raise health awareness issues:

‘Also doing the campaigns and then when people are coming in the clinic we raise awareness and also teach them about outbreaks and also to prepare for those outbreaks.’ (F4, M, P3, 26 yrs)

#### Reach educational objectives

The participants raised the point that being placed at a PHC clinic for clinical practice was to reach their educational objectives set according to the curriculum outcomes at the nursing college after the completion of clinical placement and their comprehensive 4-year course:

‘Ehh, being placed in a PHC clinic I think we are here firstly to reach our objectives that were put forward to us when we were at college, according to the syllabus and do…that at the end of the day you have, you are able to nurse a patient or diagnose and treat whatever minor ailments we find in the community.’ (F2, M, P2, 23 yrs)

#### Clinical guidance

Clinical guidance was also mentioned as another reason to be placed at the PHC clinic for clinical practice. The participants stated that there should be clinical guidance whereby professional nurses should demonstrate procedures to nursing students as well as supervise and assess whether the procedures have been undertaken correctly during the clinical practice:

‘The positive thing here is that we are working with sisters who are experienced in PHC, so what they are doing is like we are guided or mentored by them.’ (F2, M, P3, 26 yrs)

‘[*A*]nd they are independent, they don’t rely on students that much they support us, they guide us you know.’ (F3, F, P6, 24 yrs)

### Theme 2: Positive, supportive and helpful experiences at primary healthcare clinics

Experiences of clinical placement at PHC clinics were also shared, such as positive experiences related to PHC staff, but nursing students also spontaneously shared challenges with regard to staff. Further sub-themes included: environment and service-related experiences and challenges, patient-related challenges, and lack of tutor support or supervision.

#### Staff-related experiences

Professional nurses at PHC clinics were experienced as being supportive, nurturing independence, warm, welcoming, non-judgemental, skilled and, finally, encouraging the nursing students to explore:

‘They are very skilled and whenever you are making a mistake, they won’t make it a big deal, they just correct you and you will understand the mistake then.’ (F4, M, P3, 26 yrs)

Professional nurses welcomed them upon arrival at the clinics, taught them where they lacked capacity, and ensured that they reach their educational objectives:

‘When we come to the clinic for the practical part of it, they do help a lot and then they are patient with us, they don’t judge us, you know maybe educate you one day and then expect you to be knowing everything, you know it’s a continuous thing every day they help with that.’ (F4, M, P3, 26 yrs)

Professional nurses nurtured independence in the nursing students. They challenged the nursing students to explore through posing questions related to patient care and expecting them to find answers on their own by diagnosing and giving the correct treatment to the patients under supervision:

‘[… *T*]hey also allow us to be independent because at the end of the day when you are out there as a professional nurse…they want to push as much as possible for you to be able to ask questions to explore, because that is the only way you can learn.’ (F1, F, P4, 24 yrs)

This approach encouraged learning, and they were able to strengthen their clinical skills.

The participants furthermore mentioned that the staff at the PHC clinics ensured that they reach their objectives that were put forward at the nursing college according to the curriculum:

‘Personally the positive experiences that I have had in ehm, the staff here … they are always there to make sure that we fulfil school objectives that we are supposed to cover.’ (F1, F, P4, 24 yrs)

Professional nurses at the PHC clinics offered guidance and supervision during clinical practice.

#### Staff-related challenges

Whilst participants mentioned positive staff experiences, they also spontaneously shared challenges with regard to the shortage of PHC professional nurses and the heavy workload at PHC clinics:

‘I have noticed that they have a shortage of staff, they only have two PHC sisters and we are four students, so if eh it’s not easy, I think, for them to mentor us.’ (F2, F, P5, 27 yrs)

It was alluded that several staff members at PHC clinics were unsupportive. In such instances, the professional nurses treated the nursing students as if they were incompetent. Furthermore, the professional nurses were not always available to the students when they sought orientation, guidance and assistance. There was a concern that some staff members at the clinic were negligent in terms of recording patient details, ‘…and there are cards which are not filled, they are just blank’ (F3, F, P5, 24 yrs) and in the distribution of protective clothing to the nursing students such as protective face masks. ‘She gave me the N95 mask but after a week, you know’ (F3, F, P6, 23 yrs). The participants also mentioned that they noticed professional nurses absent themselves from work when nursing students are placed at the clinics.

Although the participants acknowledged that staff members are skilled and experienced, they also expressed during the interviews that more professional nurses needed clinical skills, especially PHC skills, to be able to teach and mentor nursing students and nurse patients holistically:

‘… I think it is also the shortage of staff and the skills also required by the staff, you get that certain sister has a skill that most of the rooms require but its only one sister that can help.’ (F1, F, P7, 26 yrs)

They mentioned that only a small number had been trained in PHC skills.

#### Environment and service-related experiences and challenges

With regard to the environment and services, the participants also shared both positive experiences and challenges. On the one hand, participants experienced that there was adequate availability of resources and equipment:

‘I think the availability of the equipment also, there is availability of resources to work with.’ (F4, M, P3, 26 yrs)

In contrast, the participants also mentioned that there were limited resources to perform daily procedures during patient care. Consequently, their clinical practice became so difficult that they had to buy their own equipment:

‘We also don’t have equipment, at the college they told us to buy ourselves the baumanometers, we have our own.’ (Trial run, M, P4, 28 yrs)

Students were also concerned that they could not complete all their required practical assignments because of not all services being available at PHC clinics where they were placed, for example, school health services:

‘They do not offer school health services…on that day whatever day you decide to go to school health, you can go and do that …’ (F1, F, P5, 25 yrs)

Furthermore, concerns were raised regarding sorting and triage:

‘…there must be a system in a way emergencies could be identified and not necessarily only visible emergencies because people are critically ill.’ (F2, M, P2, 27 yrs)

#### Patient-related challenges

The participants experienced that the patients showed a lack of trust and confidence towards them and preferred to be assisted by the professional nurse during consultation. They shared that some patients also displayed a negative attitude towards the professional nurses, for example, when the professional nurse recommended a new treatment regimen. The lack of compliance to chronic medication treatment by patients was also highlighted as a challenge:

‘The challenges that one is facing is with regards to the clients themselves. We have some who would not necessarily want us to attend to them and it happens the client is number one on the queue and when you call that person, the person would like to be attended by a PHC sister….’ (F2, F, P5, 23 yrs)

#### Lack of tutor support and supervision

Participants experienced that there was a lack of support and supervision from their clinical tutors during clinical placement. Nursing students expressed the concern that when they were placed at the PHC clinics, their tutors failed to either call or visit them to check on their progress:

‘With the tutors I saw that the tutors are not supportive, they never come.’ (F3, M, P7, 29 yrs)

### Theme 3: Support to cope with challenges

The participants revealed that they needed educational support, support from professional nurses at PHC settings, and service and environmental support.

#### Educational support

During the interviews, it was revealed that effective, continuous supervision by professional nurses was needed during clinical practice to help nursing students to deliver quality care. They also expressed the need to be supported and supervised by clinical tutors to achieve their educational goals:

‘[*W*]e would like to have more, you can see the clinical tutors a little bit especially maybe before the exam, you know we are going to be doing summative the following week, we would like to have the person coming in reassuring you….’ (F1, M, P8, 26 yrs)

The participants furthermore stated that clear communication between the tutors from the nursing college and the professional nurses at the PHC clinics would best support their goals to be achieved during clinical practice:

‘I think also if there can be someone specifically be allocated for students …but each time of the day there should be someone with the students, making sure that they do the exact thing.’ (Trial run, M, P6, 28 yrs)

‘Like we said before, like we have been saying, ehh like the in-charge I don’t know how can they communicate with the college but there should be clear communication between the college and the clinics that if you come and you are supposed to do 1, 2, 3, 4.’ (Trial run, F, P5, 25 yrs)

#### Support from professional nurses at primary healthcare settings

Although the participants were welcomed and felt at ease at PHC clinics, they required supportive professionals for further guidance to achieve their goals. Moreover, they viewed mutual respect between the staff members and themselves as significant as it could help them to cope with their challenges during clinical practice at PHC clinics:

‘Everyone should be treated with the same level of respect, in order for you to achieve whatever goal that you have…’ (F1, F, P5, 25 yrs)

Participants also mentioned rapport and trusting nurse-patient relationships as important aspects when providing nursing care to patients whilst placed at PHC clinics, as they needed that patients trust them during their clinical placement:

‘So I think we can solve it by health education to make them understand that even though a student or sister might be younger than them but they should regard that person as the person who would help them regardless of their age.’ (F2, F, P6, 24 yrs)

#### Service and environmental aspects

Service and environmental aspects included a need for clear communication between staff, a support system for staff and an effective rotational system:

‘In terms of communication, because at the end of the day whether you like it or not, you will have to communicate with that person because you will be serving the same patients and a report has to be given … verbal communication has to be there at some point.’ (F1, F, P6, 23 yrs)

It was also suggested that a conducive clinical learning environment included the availability of resources and equipment, school health services, an emergency identification system for patients, and separate consultation facilities for patients and dispensing:

‘I think they can order per room, each and every room to have its own equipment.’ (F1, F, P2, 25 yrs)

The availability of ward-based outreach teams and a doctor on site for needy patients were suggestions to maximise the availability of resources. Training needs included HIV/AIDS for professional nurses and the interpretation of vital signs for non-nursing staff:

‘I think workshops, we need to have workshop and look at training workshops even pre working in the morning.’ (F2, M, P4, 27 yrs)

### Theme 4: Resilience of nursing students

The respondents shared that passion for the nursing profession, personal strengths and support enabled them to be resilient.

#### Passion for the nursing profession

Most of the participants believed that they keep bouncing back from their challenges at PHC clinics because they have love, passion and pride for the nursing profession:

‘I think we have fallen in love with this profession.’ (F3, M, P1, 29 yrs)

They stated that doing noble work and making a difference in someone’s life makes them feel that they are contributing and giving back to the community:

‘It makes you feel good knowing that you have done well for someone, you know.’ (F3, M, P1, 29 yrs)

They also stated that they are encouraged by the gratitude displayed by the patients they had helped:

‘When coming across them they are all clients in the street like seeing them happy, like giving you a long Yho!!! Sister you have helped me a lot I am well now, is very fulfilling.’ (F2, F, P1, 24 yrs)

#### Personal strengths

The participants stated that their personal strengths made them resilient. They expressed their determination as a strength that motivated them to develop into professional nurses:

‘Determination, determination to reach your goal, no matter what may come we have to achieve this goal, I came here to become a professional nurse and then I will be a professional nurse….’ (F4, M, P4, 27 yrs)

Their goal to acquire skills, knowledge and experience in the nursing profession helped them to continue in spite of the challenges:

‘Personally what keeps me going is being goal-directed and knowing what I want in spite of the hardness along the road.’ (F1, F, P7, 26 yrs)

They expressed that faith in God and having confidence give them hope and strength to cope:

‘So each time I face challenges, like if this is really the journey I am supposed to go “cause one day I was like God just help me.”’ (F3, F, P3, 27 yrs)

They added that negative experiences and challenges strengthened them emotionally and spiritually:

‘And then again by having such challenges it actually strengthens you it…and it actually makes you realise how much you love your work, if you love your work even though there are challenges, storms they come and go but you still pursue.’ (F4, M, P4, 27 yrs)

Similarly, being accountable and having responsibilities and obligations motivated them to cope with difficulties during clinical practice.

#### Support

Under this sub-theme, they shared that supportive relationships boosted their confidence and increased their ability to cope during clinical practice:

‘[*A*]nd also a supportive system which is the sister who are patient with us, if they do not know, they refer back to the books and you see that the person has further interest in helping ….’ (F1, F, P7, 26 yrs)

According to the participants, support from staff members, their family members and peers helped them cope irrespective of the challenges they experienced:

‘Sometimes even ehm a good study group or just having ehm a group of peers that ehm share common goals with…’ (F3, M, P1, 29 yrs)

Positive working relationships between the staff members and nursing students during clinical practice at PHC clinics encouraged them to continue in spite of the challenges. Placement at a reputable PHC clinic for clinical practice was highlighted as a factor that helped nursing students to cope irrespective of the challenges they encountered. A reputable PHC clinic was regarded as a clinic where professional nurses were supportive, provided guidance and were willing to teach the nursing students. This also included having enough resources such as enough staff, materials and equipment:

‘… and here really we’ve got everything that we need it actually motivates us’ (F2, F, P1, 24yrs)

## Discussion

Nursing colleges have to place nursing students at PHC clinics so that they gain PHC skills and experience. Literature confirms that clinical placement in PHC creates the opportunity for effective learning because students are afforded the opportunity to practice what they have learned (Fröberg et al. 2018). The findings of this research confirmed that PHC settings can be valuable learning opportunities for nursing students who appreciated and acknowledged PHC settings as such. Motsaanaka et al. (2020) also found that nursing students learn how to deal with different patients during clinical placement. They attain a better understanding of the issues faced by patients and become more tolerant in accepting patient behaviour (Enestvedt et al. 2018). Similarly, Phafoli et al. (2018) found that being placed in a PHC setting enabled nursing students to gain a deeper understanding of the socio-cultural issues that impact the provision of PHC. Research conducted by Phafoli et al. (2018) and Mackey et al. (2018) also confirmed these findings, namely that placement in a PHC setting contributes to reaching nursing students’ educational goals.

Nursing students appreciated the involvement of professional nurses as supervisors who created an atmosphere conducive for teaching and learning by being welcoming and by challenging nursing students and encouraging their independence. In a study conducted from the supervisor, student and patient perspectives regarding student placement in PHC, supervisors also confirmed that it is their role to guide students and accompany them in their development as professional nurses (Fröberg et al. 2018). Similarly, Donough and Van der Heever (2018) reported that nursing students stated that guidance and supervision from professional nurses during their clinical placement were good.

Literature also supports the findings regarding the challenges that the participants experienced. Motsaanaka et al. (2020) found that nursing students experienced a shortage of PHC nurses and that they were regarded as workforce, that there was a lack of quality control, that professional nurses lacked appropriate qualifications such as PHC skills and that they displayed a negative attitude towards students. In another study, it was also found that nursing students did not always receive assistance and supervision from the professional nurses on duty because of the lack of staff and they had to do their best to nurse the patients (Donough & Van der Heever 2018). Phafoli et al. (2018) confirmed that nursing students experience a lack of resources at PHC settings as frustrating, but that it also stimulates their innovative thinking regarding coping with difficult situations.

Participants’ need for support is echoed in the existing literature. Similar research, conducted by Lopez et al. (2018) and Phafoli et al. (2018), confirmed this finding, namely that nursing students need support from educators, tutors and supervisors when placed in a PHC setting for clinical practice in order to facilitate deep learning.

Participants’ ability to remain resilient through having a passion for the profession, personal strengths and support was also found in the literature (Froneman, Du Plessis & Koen 2016). Robertson et al. (2016) conducted a systematic review on the resilience of PHC professionals and also concluded that having a sense of meaning and purpose contributes to resilience. Research by Phafoli et al. (2018) confirmed that nursing students experience that their confidence and competence are strengthened through being placed in a PHC setting. Robertson et al. (2016) concluded that having the strength of tolerance for and accepting uncertainty is related to a higher level of resilience. In the study conducted by Ching et al. (2020), nursing students devised ways of dealing with stress. They planned and prioritised and focused on the positive aspects of nursing rather than on the negative. Robertson et al. (2016:430) also reported that a supportive workplace environment contributes to resilience. Likewise, Kaphagawani and Useh (2013) found that an environment that positively influences learning have been reported as happy, friendly with good morale and attitude, cooperative and willing to teach and guide students to provide quality patient care.

### Recommendations

From the findings and the literature, recommendations that could be formulated are indicated in Table 2. The study’s recommendations can guide and empower professional nurses, nurse educators, nurse managers and other healthcare PHC workers to support nursing students.

**TABLE 2:** Recommendations for nursing practice to support nursing students at primary healthcare clinics.

Nursing practice	Recommendations
Support provided at PHC settings by staff and the nursing college	Professional nurses should be allocated specifically for effective and continuous supervision of nursing students during clinical practice at PHC clinics.
	Professional nurses should be prepared and motivated to fulfil the role of supervisor to nursing students. The nursing college can play an important role in such preparation.
	Nursing students should be welcomed and supported to make them feel comfortable and at ease by professional nurses.
	There should be effective rotation of professional nurses and nursing students to different programmes to improve PHC clinical skills and experience.
	Clear verbal communication should be inculcated between staff at PHC clinics and nursing students.
	Clear communication should be inculcated between PHC settings and the nursing college to clarify goals to be achieved by nursing students during clinical practice.
	Tutors should support and supervise nursing students at PHC settings, especially before clinical summative examinations.
	Professional nurses and clinical tutors should build on the strengths of nursing students to assist them to persevere in spite of challenges.
	Professional nurses and clinical tutors should conduct reflection sessions with nursing students reflecting on positive and challenging experiences and on how they managed to cope during these experiences.
Support relating to patients	Health education to patients and community should be used to inform them about the presence of nursing students at PHC clinics.
	There should be rapport and facilitation of nurse–patient relationships to improve the community’s trust in nursing students and professional nurses.
Clinical environment conducive for teaching and learning	The environment should be conducive for learning with available resources and equipment, a conducive clinical atmosphere with mutual respect among professional nurses, nursing students and the doctor.
	There should be an extended period and regular placement of nursing students at PHC clinics for clinical practice as placement is beneficial and conducive for learning.
Improvement of services and availability of resources	Managers at PHC settings should consider suggestions by nursing students to improve services and the availability of resources, which may not only improve the quality of services but also contribute towards a conducive learning environment. These suggestions may include the following:
	Consultation should be undertaken independently for the dispensing of medicines. The pharmacist should dispense medication from the pharmacy.
	An emergency identification system should be available for at risk and critical cases.
	Material resources and equipment should be available in every consultation room for easier access and use.
	School health services should be available at clinics to meet learning needs of nursing students.
	A doctor should be available daily at PHC clinics to avoid referral of patients to hospital.
	Ward-based outreach teams should be available at PHC clinics and should include home-based care for households.
Provision of training for professional nurses and non-nursing staff.	Training should be provided for PHC staff to ensure better quality services and a more conducive learning environment for nursing students. Training could include the following:
	Workshops and training for non-nursing staff to interpret vital signs.
	All professional nurses should be trained to manage HIV/AIDS and related conditions as it is the most common disease.

*Source*: Zulu, B.M., 2015, *Nursing students’ experience of clinical practice in primary health care clinics*, Masters dissertation, North-West University, Potchefstroom, viewed 17 May 2021, from https://repository.nwu.ac.za/bitstream/handle/10394/15830/Zulu_BM_2015.pdf?sequence=1&isAllowed=y.

PHC, primary health care; HIV/AIDS, human immunodeficiency virus/acquired immunodeficiency syndrome.

Regarding nursing education, it is recommended that there should be clear communication between the nursing college and PHC clinics by placing a PHC-trained professional nurse specifically for nursing students at PHC clinics. The PHC-trained professional nurse will communicate the needs of nursing students during meetings with the nursing college and get clear indications of what is required for student learning such as the students’ training file. Clinical tutors should visit nursing students regularly, especially before summative clinical examinations to provide support and guidance. Professional PHC nurses should supervise and mentor nursing students during their PHC placements. A conducive clinical environment should include a positive atmosphere where nursing students are regarded as younger colleagues. Primary healthcare clinics should be regarded as an environment conducive for learning and for correlating theory and practice.

Regarding nursing practice, professional nurses should play a major role in implementing recommendations formulated in this study (see Table 2) to enhance helpful and positive support for nursing students during clinical practice at PHC clinics and to support nursing students to cope with challenges encountered in PHC settings. Support should be provided at the PHC settings by staff and the nursing college, including support relating to patients and to the clinical environment conducive for teaching and learning; there should be improvement of services and availability of resources and the provision of training for professional nurses and non-nursing staff members.

Further research is needed on the implementation of recommendations for nursing practice and education to support nursing students to cope with challenges during PHC clinical practice in all regions of the country.

### Limitations

Although this study obtained rich information about the experiences of nursing students when allocated at PHC clinics for clinical practice, the following limitation was identified:

Semi-structured focus group interviews which were scheduled to take place at the nursing college, could not take place because of the little time allocated (2 weeks) for theory. In the process, the mediator and the first author reached an agreement that interviews should be conducted at PHC clinics where nursing students had been placed. Some students who were willing to participate were placed in other regions and provincial clinics which did not belong to the region used in this study. Consequently, they could not be interviewed as they fall under the exclusion criteria. Some nursing students who were willing to participate were placed at distant clinics and could not be interviewed.

## Conclusion

The study revealed that nursing students encountered negative and positive experiences at PHC clinics. Both experiences confirmed that PHC settings can be valuable learning opportunities for nursing students who appreciated and acknowledged PHC settings as such. Nursing students appreciated the involvement of professional nurses as supervisors who created an atmosphere conducive for teaching and learning by being welcoming and through challenging nursing students and encouraging their independence. The study’s recommendations can guide professional nurses, nurse educators, nurse managers and other healthcare workers to support nursing students and strengthen students’ resilience during these clinical challenging situations. These include support that should be provided at PHC settings by staff and the nursing college, including support relating to patients and to the clinical environment conducive for teaching and learning, there should be improvement of services and availability of resources and the provision of training for professional nurses and non-nursing staff members. As such, the study recognised the resilience of nursing students when placed in PHC clinics for clinical practice and supervision.
